# The Different Muscle-Energetics during Shortening and Stretch

**DOI:** 10.3390/ijms12052891

**Published:** 2011-05-03

**Authors:** Robert Jarosch

**Affiliations:** Formerly Institute of Plant Physiology, University of Salzburg, Hellbrunnerstrasse 34, A-5020 Salzburg, Austria; E-Mail: ilse.foissner@sbg.ac.at; Tel.: +43-07612-67972; Fax: +43-662-8044-619

**Keywords:** active and passive thin filament-rotation, frictional heat

## Abstract

The helical shape of the thin filaments causes their passive counterclockwise rotation during muscle stretch that increases tensile stress and torque at first by unwinding and then by winding up the four anchoring Z-filaments. This means storage of energy in the series elastic Z-filaments and a considerable decrease of the liberated energy of heat and work to (h—w_ap_), where h is the heat energy and w_ap_ the stretch energy induced from outside by an apparatus. The steep thin filament helix with an inclination angle of 70° promotes the passive rotation during stretch, but impedes the smooth sliding of shortening by increased friction and production of frictional heat. The frictional heat may be produced by the contact with the myosin cross-bridges: (1) when they passively snap on drilling thin filaments from cleft to cleft over a distance 2 × 2.7 nm = 5.4 nm between the globular actin monomers in one groove, causing stepwise motion; or (2) when they passively cycle from one helical groove to the next (distance 36 nm). The latter causes more heat and may take place on rotating thin filaments without an effective forward drilling (“idle rotation”), e.g., when they produce “unexplained heat” at the beginning of an isometric tetanus. In an Appendix to this paper the different states of muscle are defined. The function of its most important components is described and rotation model and power-stroke model of muscular contraction is compared.

## 1. Introduction

In their basic work on the “Energetic Aspects of Muscle Contraction” Woledge, Curtin and Homsher ([1], p. 209) describe the effect of stretch: “When an active muscle is stretched, its mechanic and energetic behavior is strikingly different from that during shortening or in isometric contraction and, after the end of the period of stretching, differences persist”. For definitions of “active muscle”, *etc.*, see the Appendix.

After a survey on the historical observations of the energetic differences between contracting and stretched muscles the following pages analyze the possible reasons for the differences.

This small paper is a supplement to my larger one on the mechanics of muscular contraction on the basis of filament rotations [2], where these reasons so far have been briefly mentioned. It is advantageous for the reader to first study the larger paper [2] to recognize the possible reasons for the filament rotation and its importance for the general muscle mechanics. However, a summary of these subjects is also given in the Appendix to this paper with definitions of the different states of muscle, the function of its most important components, and a comparison between rotation model and power-stroke model of muscular contraction.

## 2. Historical Survey

First energetic investigations were done in 1864 by Heidenhain [3] “who concluded from some rather simple experiments with elementary instruments there available that the energy liberated by a stimulated muscle depends on its length, on the change of length during contraction, on the load lifted, and so on the mechanical work done” (quot. Hill [4], p. 145). About twenty years later Fick [5,6] determined the “total energy of a contraction as the algebraic sum of mechanical work and heat produced by the muscle. This quantity was found to be smaller in the case of a stretched muscle and no satisfactory explanation was available” (quot. Levin and Wyman [7], p. 218). During his life-time A. V. Hill worked expertly on the improvement of the heat-measurement and was very successful (in 1922 Nobel award, in 1926 first demonstration of the extremely small heat production during nerve excitation [8]). In 1965 in his comprehensive book [4] he gives a wealth of information on the physiology of muscle and nerve with a summary of his life-work and a detailed description of his numerous papers also with coworkers. Hill’s coworker did important work using his improved techniques. “Fick’s heat measurements have been substantiated by the researches of Fenn [9,10] and Wyman [11]. Wyman found, just as Fick had done, that the muscle liberated more total energy when it shortened and did external work than when it was stretched and work was done on it” (quot. Levin and Wyman [7], p. 220). “Fenn’s main conclusions were: (a) that when a muscle shortens, and does mechanical work, extra-heat is liberated; and (b) that when a muscle is forcibly lengthened during contraction (so that the work it does is negative) the extra energy is negative too” (quot. Hill [4], p. 145). Hill designated work and heat of shortening as the “positive Fenn effect” and absorption of energy during the negative work of stretch as the “negative Fenn effect” ([4], p. 149). Further papers on later experiments on the absorption of work during stretch were discussed ([4], p. 150). When stretch work is done on the muscle by an apparatus (w_ap_) the liberated energy during stretch (h—w_ap_) is reduced. The reduction is small when slow isotonic stretches are applied [12] but becomes significant by quick isovelocity stretches [13]. A quick isovelocity stretch of 50% V_max_ can reduce the energy to less than 30%, or it becomes negative, but the heat production can increase over the isometric rate (Abbott *et al.* [13], Hill and Howarth [14]). The latter authors assumed that during stretch the work is absorbed in reversing the driving chemical reactions provoked by the stimulus. But they asked alternatively: “Could the mechanical energy which disappears be temporally stored in some form from which it is slowly released as heat between one contraction and the next…?” It was further considered that the series elastic component of muscle was the only component that could store mechanical energy. Woledge *et al.* ([1], p. 215) discussed three suggestions for the stretch-dependent decrease in the rate of production of (h—w_ap_):

1. The processes that produce the heat during isometric contraction are prevented by the stretch.
2. These processes are not just prevented but actually reversed.
3. An extra endothermic process occurs during stretch. An example of such a process would be the storage of work in a mechanically strained structure. They conclude that (1) occurs, but that (2) does not occur. This leaves (3) as the most likely explanation of the negative rate of (h—w_ap_). They noticed “that Cavagna and Citterio [15] and Edman *et al.* [16] have suggested, on the basis of mechanical observations, that work can be stored in muscle in this way”. Recently, Linari *et al.* [17] provided detailed quantitative data concerning the amount of the stored energy.

## 3. The Underlying Mechanics

The thin filaments of muscle are protein double helices with right-handed coiling sense. When they move against the myosin filaments they must follow the rules of the mechanics valid for helical bodies: During stretch of contracting muscle, the thin filament helices are drawn out off the myosin filaments and their cross-bridges and must passively rotate in counter-clockwise direction, as seen from the Z-bands. This can be easily demonstrated by large scale models [2,18,19]. Since each thin filament is anchored by four Z-filaments tensile stress and torque increase during stretch by unwinding and winding up the Z-filaments. This appears in the electron microscope as a change from the “small square” to the “basket weave” pattern [20]. The outer energy of stretch w_ap_ is brought into the muscle and reduces the value of (h—w_ap_). The muscle tension can strongly increase, e.g., to the double value of the isometric tension and is stored, as was considered by Hill and Howarth [14] and more founded in details by Cavagna and Citterio [15], Edman *et al.* [16] and Linari *et al.* [17], as described above. Without the knowledge that the Z-filaments are wound up by the passive counter-clockwise rotation of the thin filaments during stretch, the increased force was presumably erroneously interpreted, e.g., by Brunello *et al.* [21] by an increased number of cross-bridges attached to actin during stretch.

To understand the actomyosin and ATP-kinetics one should include the filament rotation into the theory. Already Justus v. Liebig suggested in 1839 a dynamic principle for the catalytic activity. Accordingly, the catalyst transmits its own movements to the substrate (quoted from Needham ([22], p. 31). The clockwise drilling rotation of the thin filaments in shortening muscle may be supported by winding up and unwinding of myosin chains, e.g., the α-helical light chains, which take contact with the thin filament during contraction ([23] p. 285, see also p. 160 and p. 400 of this book). When during stretch of a shortening muscle the direction of the thin filament rotation is reversed, it follows that, e.g., unwinding of a myosin chain from the thin filament is also stopped or reversed to winding up. Thus, an ATP molecule bound by the myosin chain and with actin, when wound up on the thin filament by stretch, could be torn during the unwinding process of shortening. I thank one of the reviewers for his indication to the work of Curtin and Davies [24], showing that the rate of ATP-splitting is much decreased or ceased, during muscle stretch.

During release and muscle shortening the stored tensile stress and torque can be used for doing work by clockwise drilling rotations. Moreover, the shape of the thin filament helices must be very important for the different sliding behavior and the energetics of stretch and shortening. The large inclination angle (about 70°, see Figure 2 in [2]) of the thin filament helix should promote the passive counterclockwise rotation during stretch with no or little slippage, but should cause increased resistance during the active clockwise drilling rotation of shortening (more frictional heat production and cross-bridge slippage). Presumably the promotion of stretch rotation is especially important on passive muscle, where slack can be removed by the stretch [2].

In the long history of muscle research (about 200 years without clarification of its mechanism), old conceptions have been forgotten since they were not seen in a causal connection, for example the heat-production of muscle by friction. Hill and Hartree [25] still have written: “In a rapid shortening muscle a large part of the potential energy is wasted and can only reappear as heat… If the change be very slow indeed, little energy is lost by internal friction; if, however, the change be rapid, the loss of energy… may become large and lead to a considerable production of heat”. And Lupton [26]: “In the muscle of the human arm a movement completed in 1 second wastes 26% of its energy in overcoming the frictional resistance of the muscle itself…”.

The frictional heat produced in the overlap region of the thin and thick filaments is the most spectacular heat fraction during shortening, relaxation and in the isometric stage. The overlap-independent “activation heat” and “recovery heat” depend on Ca and metabolic reconstitution processes (for definition see [1]).

The frictional shortening heat of muscle is analogous to the frictional heat produced by a drilling machine. The amount of heat increases with the rotation velocity of the borer, respectively, with the shortening velocity of muscle.

During shortening near V_max_ most frictional heat is produced, and energy-balance studies show a significant discrepancy between the observed and the explained enthalpy that decreases with the shortening velocity. The discrepancy was not found more in shortening at ½ V_max_ [27].

How is the frictional heat being produced by the contact of myosin cross-bridges with the rotating thin filaments? This may depend on the type of rotation: (a) A pure forward drilling rotation is performed, e.g., by a cork-screw in the cork-stopper. (b) A helix rotating on the same place (no forward drilling at all = “idle rotation”) shows it turns as helical waves running into the opposite direction of forward drilling. *In vitro* sliding actin filaments can perform this type of rotation when they “wiggle” and slip against the substratum. (c) Both motions, forward drilling and helical waves exist at the same time, e.g., during usual drilling in wood. The tips of the thin filaments may show this rotation type. But the situation in muscle is more complicated. Since the torque produced by tropomyosin (supported by myosin) propels the thin filament rotation, the rotation velocity of the free tips should be maximal and should decrease with the distance to the Z-band. Therefore, the sliding velocity of shortening should be maximal, when only the distal parts of the thin filaments are in contact with the myosin cross-bridges. When the filaments show large overlap, the sliding velocity should be a mean value of the different rotation velocities of the sites in contact with the cross-bridges. All cross-bridges, in contact with quicker or slower rotation velocities than the mean value, must slip. Presumably, this explains the rather constant velocity of unloaded shortening in the range of sarcomere length 1.65–2.7 μm (for more details, see [2]).

During sliding the myosin cross-bridges can contact the globular actin monomers in the grooves of drilling thin filaments. The distance of the actin monomers in one groove is 5.4 nm (2 × 2.7 nm). The cross-bridges may passively snap from one cleft between the monomers to the next, causing the stepwise shortening of sliding (see Pollack *et al.* [28]). This motion should be similar during stretch and shortening. When the shortening velocity is quick (V_max_), the cross-bridges may jump over two or more monomers, so that the frictional heat can decrease with higher velocity. Hill [29] found decreased heat during high shortening velocities.

The cross-bridges must take a different contact with the thin filaments during their passive cycling, when the thin filaments do not drill but rotate on the same place (“idle rotation”). As described above, the turns of the helical filaments run here to the Z-band as helical waves, causing the slippage and passive cycling of the cross-bridges. They can cycle over a distance of 36 nm (the half pitch) from one helical groove to the next. Presumably this motion occurs at the beginning of an isometric contraction, when only a few cross-bridges are present, that may produce frictional heat as an “unexplained energy” [2]. When the idle rotation turns to drilling, both motions, drilling and opposed waves are present. The cycling velocity of the cross-bridges should decrease with the portion of drilling, and may become the short stepwise motion, in case that pure forward drilling is attained.

Fenn [9,10] found that during shortening more heat is produced than in the isometric stage. The steady isometric stage may be described as a dynamic balance between the torque in the thin filaments plus Z-filaments, and the load-dependent resistance of the overlap cross-bridges, that allows only a fine oscillating drilling rotation of small amplitude, which is visible with a good resolution of the tension curve. Here, the cross-bridges may snap from cleft to cleft between the actin monomers, as described. The extra-heat of shortening should depend on slipping and cycling cross-bridges.

Linari and Woledge [30] described experiments with shortening fibers showing differences in heat production depending on whether shortening was constant during a given length, or performed by a series of steps (staircase shortening). A series of steps should produce more friction since the redeveloped force after each quick release depends on additional internal rotation during rewinding the Z-filaments [2].

Finally the old results of Blix [31] should be mentioned, who always found the highest heat rate in the isometric stage, and that the muscle length promotes it too (“Länge macht Wärme”). It is possible that Blix with his simple thermo-galvanometer measured the heat which was later described as the “unexplained energy”: When an elongated muscle with little overlap is clamped, the thin filaments cannot drill after activation, since the muscle is clamped. But they may perform idle rotation with slipping and cycling cross-bridges that can produce heat as in the case of the “unexplained energy” at the beginning of an isometric tetanus.

## Appendix

### Definitions of Muscle States and Function of the Most Important Components

Active muscle is a muscle activated by a stimulus (a nerve impulse or electric shock). Ca^2+^ becomes free from the sarcoplasmic reticulum, is bound by the muscle filaments, and activates the filaments.

Contracted muscle. The contractile component of muscle, the thin filaments rotate and slide against the thick myosin filaments and their cross-bridges which have been drilled out from the thick filaments by the Ca^2+^ activation. This causes either shortening (isotonic contraction) or a strong force (isometric stage of contraction), when shortening is fully prevented by a heavy load or by clamping the muscle ends. The force arises by stretching and twisting around the four elastic Z-filaments, which anchor each thin filament. The Z-filaments are the most important series elastic component of muscle. Shortening and force-generation takes place, when the muscle can lift its load.

Released muscle. When a muscle in the isometric stage is released, it shortens (isotonic contraction) and loses its force for a short time. After shortening is stopped, the force arises again as the “redeveloped force”. The redeveloped force decreases (“force deficit”) with increasing release distance.

Stretched muscle. Active muscle resists strongly against stretch. The stretch produces additional force (“stretch activation”) that can exceed the maximal isometric force when the thin filaments are passively rotated and wind around the four Z-filaments.

Resting muscle (or passive muscle) is a non-activated muscle that easily can be elongated by stretch. But stretch can produce strong force also on non-activated muscles when thin filaments are rotated.

### A Molecular Mechanism for the Active Rotation of the Thin Filaments

Since a reviewer asked general questions about the rotation model, a short survey is given here, that may be interesting for other readers. Indeed, the theory of the rotating thin filaments is proved by many facts. The most simple is a pure mechanical one: The thin filament helix cannot move against many adjacent cross-bridges without rotation, as a corkscrew cannot move in the cork-stopper without rotation. It was shown in my larger paper [2], that the assumption of rotating thin filaments leads to a solution of the main problems of muscle physiology, *i.e.*, stretch activation = force-enhancement after stretch, force-deficit after abundant shortening, force regulation by Ca^2+^-binding or Ca^2+^-displacement by the four twisting Z-filaments, Fenn-effect, latency-relaxation by unwinding of the four Z-filaments, *etc.* The accordance with so many facts may be a quality of a correct theory! I cannot not repeat all the facts in this paper, but here I will repeat the probable molecular mechanism for the active rotation of the thin filaments: Rotating filaments and presumably all the active motility of life, depend on proteins that contain α-helical chains with strongly charged side-chains, which can repel each other in axial direction. When these side-chains interact with ions, the repulsion decreases, and the α-helical H-bonds shorten cooperatively. This means a small pitch-decrease of the α-helix and a generation of torque and torsional rotations [2,32]. The two long coiled coils of tropomyosin in the two grooves of the thin muscle filaments can generate more than 30 rotations after Ca^2+^-binding to troponin, that also changes the conformation of tropomyosin. Further torque is produced by α-helical myosin chains which may be wound up by the rotating thin filaments. The generated torque twists the actin filament, so that its free tip can rotate. The twist of the thin filament can be recognized by the position of the troponin pairs, situated along the thin filament in a distance of 38.5 nm. These troponin pairs do not lie on a straight line parallel to the thin filament axis but on a slowly twisting helix ([33], p. 52), that should vary during the thin filament rotation.

### The Rotation Model Compared with the Power-Stroke Model of Muscle Contraction

A reviewer asks in which sense the contractile component works differently from conventional power-stroke models.

- The rotation model explains filament sliding much simpler. The contractile component of muscle, the thin filaments are solid protein helices, which can rotate by torque, as described above. Torque and tensile stress is stored by the elastic Z-filaments, the series elastic component of muscle. Since four Z-filaments anchor each thin filament, they are untwisted or twisted by the thin filament rotation. The sliding process between thin and thick filaments that results in muscle shortening, is the consequence of a single mechanical process, namely the clockwise rotation of thin filaments when they drill into the myosin filaments and their adjacent cross-bridges.
  On the other side, the power-stroke model of myosin cross-bridges needs four succeeding conformational changes to explain the sliding process: (1) A conformational change of the myosin S_2_ part to attach the thin filament. (2) A conformational change to perform the power stroke in the attached S_1_ part, the so-called lever arm motion. (3) Detachment by a conformational change again of the S_2_ part. (4) A conformational change in S_1_ for the recovery stroke of the lever arm. Mechanics and control of these processes are unknown. The recent experiments of Sugi *et al.* [34], using a hydration chamber in the electron microscope, were interpreted as the recovery-stroke of cross-bridges. I thank Prof. Sugi for his kind sending of a most recent paper [36], still being in press. If the power-strokes of cross-bridges really exist, they can propel or support only the thin filament drilling rotation, since the helical filaments are embedded within many cross-bridges, like a cork-screw in the cork. Another displacement than drilling is not possible by mechanical reasons! Also a lateral component of the power-strokes is here not necessary. When the power-strokes push in axial direction, the high inclination angle (70°) of the thin filament helix should promote its drilling motion, as it is promoted in opposite direction by muscle stretch (see Chapter 3). The power-strokes may attack target zones for myosin binding, as assumed for in vitro gliding and twirling actin filaments [35]. When the thin filament rotation is supported by myosin cross-bridges, both rotation model and power-stroke model would be correct – a surprising solution of the original antithesis!
- In the rotation model the regulation of muscle force is determined by the load of muscle. The load determines a certain twisting stage of the four series elastic Z-filaments and at the same time the amount of Ca^2+^-binding to the Z-filaments, or Ca^2+^-displacement as the “extra Ca^2+^”. The Ca^2+^-binding is maximal in the isometric stage.
  How the power strokes only (without thin filament rotation) can regulate the muscle force, is not clear. One can assume that velocity and frequency of the power strokes determine the shortening velocity. But how force, velocity and frequency of the power strokes are controlled, remains obscure.
- The rotation model explains the increased force after stretch (stretch activation) by increased torque and tensile stress in the thin filaments and in the Z-filaments after the counterclockwise rotation of the thin filament helices during stretch. The “springs” are wound up by stretch. A large shortening of muscle produces force deficit that is scanty force, because the abundant clockwise thin filament rotation decreases torque and tensile stress excessively.
  A plausible explanation of stretch activation and force deficit is difficult without filament rotation.
- The rotating thin filament helix can also produce passively cycling cross-bridges. Myosin cross-bridges that lock into clefts or grooves of the rotating thin filament helix can generate friction and frictional heat. At the same time power stroke-like motions may be produced either during drilling, when they snap from cleft to cleft between the actin monomers (distance 5.4 nm) in the grooves of the thin filament, or when the thin filament rotates on the same place (“idle rotation”, without drilling), where the cross-bridges can snap from one helical groove to the next (distance about 36 nm).
  Active processes as the torsional rotation of α-helices must occur also in the activated myosin filaments. An active rotation of the lever arm was assumed as the molecular basis for the power-stroke.

### Correction

In my paper [2] p. 2673, last paragraph, and p. 2674, second paragraph, it was erroneously quoted that increased spacing between the thin filaments was found in the isometric stage. This is wrong: The distance between the thin filaments decreases in the isometric stage!
